# Proteome Analysis of Poplar Seed Vigor

**DOI:** 10.1371/journal.pone.0132509

**Published:** 2015-07-14

**Authors:** Hong Zhang, Wei-Qing Wang, Shu-Jun Liu, Ian Max Møller, Song-Quan Song

**Affiliations:** 1 Key Laboratory of Plant Resources, Institute of Botany, Chinese Academy of Sciences, Beijing, China; 2 Department of Molecular Biology and Genetics, Aarhus University, Flakkebjerg, Slagelse, Denmark; Zhejiang University, CHINA

## Abstract

Seed vigor is a complex property that determines the seed’s potential for rapid uniform emergence and subsequent growth. However, the mechanism for change in seed vigor is poorly understood. The seeds of poplar (*Populus* × *Canadensis* Moench), which are short-lived, were stored at 30°C and 75±5% relative humidity for different periods of time (0–90 days) to obtain different vigor seeds (from 95 to 0% germination). With decreasing seed vigor, the temperature range of seed germination became narrower; the respiration rate of the seeds decreased markedly, while the relative electrolyte leakage increased markedly, both levelling off after 45 days. A total of 81 protein spots showed a significant change in abundance (≥ 1.5-fold, *P* < 0.05) when comparing the proteomes among seeds with different vigor. Of the identified 65 proteins, most belonged to the groups involved in metabolism (23%), protein synthesis and destination (22%), energy (18%), cell defense and rescue (17%), and storage protein (15%). These proteins accounted for 95% of all the identified proteins. During seed aging, 53 and 6 identified proteins consistently increased and decreased in abundance, respectively, and they were associated with metabolism (22%), protein synthesis and destination (22%), energy (19%), cell defense and rescue (19%), storage proteins (15%), and cell growth and structure (3%). These data show that the decrease in seed vigor (aging) is an energy-dependent process, which requires protein synthesis and degradation as well as cellular defense and rescue.

## Introduction

By definition, seed vigor is the sum of those properties that determine the activity and performance of seed lots of acceptable germination in a wide range of environments, including the rate and uniformity of seed germination and seedling growth, the emergence ability of seeds under unfavourable environmental conditions, and the performance after storage, particularly the retention of the ability to germinate [1]. The vigor of a seed or a seed population is, in effect, a measure of the extent of damage that has accumulated as viability declines [2]. Because high vigor is a prerequisite for an efficient stand establishment and high crop yield, it has important economic and ecological consequences. The ability of seeds to persist in soils also influences the population dynamics of ecosystems, and the duration that seeds remain viable in a gene bank determines the cost of their conservation as well as the ability of curators to maintain the genetic identity of the sample [2].

The development of orthodox seeds can be divided conveniently into three different stages, histodifferentiation, maturation and maturation drying [3]. Some authors have argued that seeds attain maximum germinability at the end of the seed-filling phase and then age [4], others have proposed that storability, which can be considered as an indicator of vigor, improves during maturation drying, the last stage of seed development where the water content decreases [5–7]. Loss of seed vigor, and subsequently of seed viability, is highly dependent on temperature and seed moisture content [8]. A decrease in seed vigor, which mimics natural aging, can be induced experimentally by exposing seeds to a high relative humidity (RH) and high temperature [9,10]. Seed vigor can also be affected by priming, a technique involving short-term imbibition followed by redrying, which improves the germination and emergence rate and decreases the seed sensitivity to external factors [11,12].

Free radicals and lipid peroxidation are widely considered to be major contributors to the decrease in seed vigor (aging), including loss of membrane integrity, reduction of energy metabolism, impairment of RNA and protein synthesis, and DNA degradation [8,13–16]. Rajjou et al. [10] compared the biochemical behavior of seeds submitted to controlled aging treatment (CAT) and to natural aging, and found some common changes in abundance of β-mercaptopyruvate sulfurtransferase (MST) and 60S ribosomal protein, in oxidation patterns of the seed proteome and in repression of translational capacity during both CAT and natural aging.

Proteome analysis is an important tool that can be used to compare complex mixtures of proteins and gain a large amount of information about the individual proteins involved in specific biological response and/or process [10,17–21]. Moreover, proteomics offers an opportunity to examine simultaneous changes in, and to classify temporal patterns of, protein accumulation occurring in complex developmental processes [22–25]. Some proteomic analyses of seed vigor change have been reported [10,17,21,24,26–30]. Although these reports have provided a large amount of information on seed vigor, the key events (proteins) associated with seed vigor change are still poorly understood.

The *Populus* genus includes a number of common species dominating the riparian woodland ecosystem in the Northern hemisphere [31]. Poplar is also fast-growing and it is therefore widely used for biofuel production [32]. A single mature female *Populus* can produce thousands or even millions of cottony seeds in most years [33], but the recruitment of new individuals is rare in the natural environment [34]. The longevity of *Populus* species seeds is very short and is limited to a few days or weeks under natural conditions [31,35–37]. Wang et al. [38] developed a threshold model to quantify the effects of temperature, storage time and their combination on germination of poplar seeds. However, the mechanism of poplar seed vigor change (aging) is not clear. The genome of *Populus* has been sequenced [39], making it a convenient species to study using proteomics. In the present study, mature poplar seeds were subjected to CAT (storage for different periods of time at 30°C and 75% RH) to obtain the seeds with different vigor. We monitored proteome changes associated with seed vigor to understand the molecular mechanism and identify new markers of seed vigor.

## Materials and Methods

### Ethics statement

No specific permits were required for the described field studies. The location is not privately-owned or protected in any way, and the field studies did not involve endangered or protected species.

### Seed collection and drying

The period of poplar (*Populus × canadensis* Moench) seed dispersal was from 25 April to 20 May, and the peak time was between 1 and 15 May in 2012 in the Beijing Botanical Gardens (N 39°59', E 116°139'; altitude, 73 m), Xiangshan, Beijing, China. The mature poplar seeds with cotton were collected on 12 May, 2012. After collection, the cotton on the seed surface was manually removed, and seeds without cotton were dried at 28±2°C and 75±5% RH for 4 d. When the water content of seeds reached 9.9%, the seeds were used as experimental material.

### Water content determination

Four replicates of 100 seeds each were sampled for water content determination according to the International Seed Testing Association [1]. Seed water content is expressed in % (w/w) of fresh weight.

### Controlled aging treatment of seeds

To obtain different vigor seeds, three replicates of 5000 seeds each were stored under controlled aging condition at 30°C and 75±5% RH for 0–90 d. After that, the seeds were used for germination, relative ion leakage, respiration and proteomic analysis.

### Seed germination

Three replicates of 100 seeds each were incubated on two layers of filter paper moistened with 3 ml of distilled water in closed 60-mm-diameter Petri dishes in darkness at 10, 15, 20, 25, 30, 35 and 40°C, respectively. Distilled water was added to the filter paper each day to maintain constant moisture during germination. Radicle protrusion to 1 mm was used as the criterion for completion of germination. The germination test was stopped when no germination of seeds was observed within 7 d.

### Measurement of relative leakage of electrolyte

After CAT for different periods of time, 50 seeds were placed in a centrifuge tube with 20 ml distilled water and conductivity was immediately measured (A_0_) using a conductivity instrument (DDSJ-308F, Shanghai, China). The centrifuge tube with seeds was placed at 25°C for 6 h during which it was shaken, and the conductivity was measured (A_1_) again. Finally, the centrifuge tube was placed in boiling water for 1 h, and was then cooled to 25°C, and final conductivity was measured (A_2_). The relative leakage was calculated as (A_1_–A_0_)/(A_2_–A_0_)×100%.

### Measurement of respiration rate

The respiratory rate of poplar seeds exposed to CAT for different periods of time was measured at 25°C using a calibrated oxygen electrode (OXYTHERM, Hansatech, King's Lynn, UK). The reaction medium contained 50 mM potassium phosphate (pH 7.2) and 0.1 M sucrose. The O_2_ concentration in the air-saturated medium was taken as 250 μM. The respiratory rate was corrected for the oxygen consumption by the electrode, and the results are expressed as nmol O_2_ min^-1^ g^-1^ DW.

### Preparation of protein samples

Poplar seeds exposed to CAT for 0, 45 and 90 d, respectively, were imbibed for 2 h at 10°C in water. Three replicates of 100 seeds (0.5 g) from each of the three treatments were homogenized in 1.5 ml precooled extraction buffer composed of 50 mM Tris-HCl (pH 7.5), 30% (w/v) sucrose, 10 mM ethylene glycol-bis-(β-aminoethylether)-N,N,N’,N’-tetraacetic acid, 1 mM phenylmethanesulfonyl fluoride, 1 mM dithiothreitol (DTT), and 1% (v/v) Triton X-100. After homogenization, the homogenate was transferred to a 10 ml centrifuge tube and the mortar was washed with 0.5 ml extraction buffer. Finally, the total of 2 ml homogenate was centrifuged at 16 000 g for 10 min at 4°C, and the supernatant was centrifuged at 32 000 g for 20 min at 4°C. The resulting supernatant was mixed with two volumes of ice-cold Tris-HCl (pH 7.5)-saturated phenol and shaken on ice for 30 min. After centrifugation at 16 000 g for 20 min, the phenol phase was collected and 5 volumes of precooled methanol saturated with (NH_4_)_2_SO_4_ was added to precipitate the proteins by an overnight incubation at –20°C. The pellets were rinsed four times with ice-cold acetone containing 13 mM DTT, and then lyophilized. The protein concentration was determined according to the method of Bradford [40] using bovine serum albumin as the standard.

### Two-dimensional (2-D) gel electrophoresis

Isoelectrofocusing (IEF) was performed mainly as described in reference [25]. Major changes were that 600 μg protein sample was dissolved in 300 μl sample buffer containing 7 M urea, 2 M thiourea, 2% (w/v) CHAPS, 20 mM DTT, and 0.5% (v/v) immobilized pH gradient (IPG) buffer (pH 5–8) and was loaded onto the strip. The gel strip was incubated at 20°C for 16 h and then used for IEF. IEF was performed by applying a voltage of 250 V for 1 h, ramping to 500 V over 1 h, 2 000 V for 2 h, 10 000 V for 4 h and holding at 10 000 V until a total of 60 kVh was reached. Prior to the second dimension, the gel strips were reduced for 15 min with 65 mM DTT in 3 ml of equilibration buffer (6 M urea, 30% (v/v) glycerol, 2% (w/v) SDS, 50 mM Tris-HCl (pH 8.8), 0.01% (w/v) bromophenol blue) and then alkylated with 2.5% (w/v) iodoacetamide in the same buffer for 15 min. After equilibration, the strips were applied to vertical SDS-polyacrylamide gels (12% resolving and 5% stacking), and the low-molecular-range markers (Bio-Rad) were loaded at one end of the strip before sealing with 0.5% (w/v) low-melting agarose in SDS buffer containing 0.01% (w/v) bromophenol blue. After solidification of the gel, electrophoresis was performed at 15°C in SDS electrophoresis buffer (pH 8.3), containing 25 mM Tris base, 192 mM glycine and 1% (w/v) SDS, for 30 min at 25 mA and for 4 h at 40 mA. The gels were stained overnight with 0.25% (w/v) Coomassie brilliant blue R-250 (CBB) in 5:1:4 (v/v) methanol: acetic acid: water and destained with 2:1:7 (v/v) methanol: acetic acid: water solution with 3–5 changes of the solution, until a colorless background was achieved.

### Image analysis, in-gel digestion with trypsin and protein identification by MALDI-TOF-TOF mass spectrometry (MS)

Image analysis of protein spots was carried out according to the method of Huang et al. (23) with a little modification. The 2-D gels were scanned at a 300 dpi resolution with a UMAX Power Look 2100XL scanner (Maxium Tech, Taipei, China). Spot detection and gel comparison were made with ImageMaster 2D Platimum (version 5.01, GE Healthcare Bio-Science, Little Chalfont, UK). After automated detection and matching, manual editing was carried out to correct the mismatched and unmatched protein spots.

Well-separated gels of the three independent biological replicates were used for proteomic comparison. The spots were considered reproducible when they were well resolved at least in two biological replicates. The normalized volume of each spot was assumed to represent its protein abundance. When comparing spot size between groups, a difference was considered significant when the change was >1.5-fold and *P* < 0.05 (t-test).

Differentially accumulated protein spots were excised from the stained gels. In-gel digestion and peptide extraction were performed as described by Wang et al. (25). Major changes were as follows: gel spots cut into small pieces were washed and destained using a series of washes consisting of 100 μl water followed by 100 μl 50% (v/v) acetonitrile (ACN) (Fisher Scientific, Fair Lawn, NJ, USA) until the blue dye was removed. Destained gel pieces were dehydrated by 15 min incubation with 50 μl 100% ACN at 25°C. For protein reduction and alkylation, the samples were first incubated with 10 mM DTT in 100 mM NH_4_HCO_3_ for 45 min at 56°C, followed by incubation with 55 mM iodoacetamide in 100 mM NH_4_HCO_3_ for 30 min at 25°C in darkness. After a series of washes mentioned above, gel pieces were dehydrated again in 50 μl of 100% ACN. Samples were subsequently rehydrated in digestion buffer (10 ng trypsin in 50 mM NH_4_HCO_3_) for 45 min on ice. After rehydration, the excess of digestion buffer was discarded, the gel pieces were covered with 50 mM NH_4_HCO_3_ and incubated overnight at 37°C.

Prior to MS analysis, peptide mixtures were desalted on in-house made microcolumns packed with reverse phase material POROS 20R2 (Applied Biosystems, Carlsbad, CA, USA). After washing with 10 μl of 1% (v/v) trifluoroacetic acid (TFA), peptides were eluted from the column with 3 μl matrix solution (5 μg/μl α-cyano-4-hydroxycinnamic acid in 70% (v/v) ACN and 0.1% (v/v) TFA) directly onto the MALDI target plate (Opti-TOF 384 Well Insert, Applied Biosystems). MS and MS/MS spectra were acquired in positive reflector ion mode by a 4800 Plus MALDI TOF-TOF MS (Applied Biosystems). The MS spectra in the range of 700–3500 m/z were acquired automatically with external calibration to a standard β-lactoglobulin tryptic digest. Collision-induced dissociation was used for fragmentation of the 10 most intense precursor ions and was performed automatically with default calibration. Data Explorer Software (Applied Biosystems) was used to convert MS and MS/MS spectra to peak lists. These were then merged into mgf files using an in-house generated script. The mgf files were used to search protein databases using Mascot (Matrix Science, Boston, MA, USA). The following search parameters were used: Database–NCBInr and/or SwissProt, Taxonomy–green plants, maximum 1 missed cleavage, cysteine carbamidomethylation as a fixed modification, methionine oxidation and N-terminal acetylation as variable modifications, mass tolerance of 70 ppm in MS mode and 0.6 Da for MS/MS. Protein scores greater than 58 in Swissprot database or 73 in NCBInr database were significant (*P* < 0.05).

## Results

### Changes in poplar seed vigor during aging

**Fig 1:**
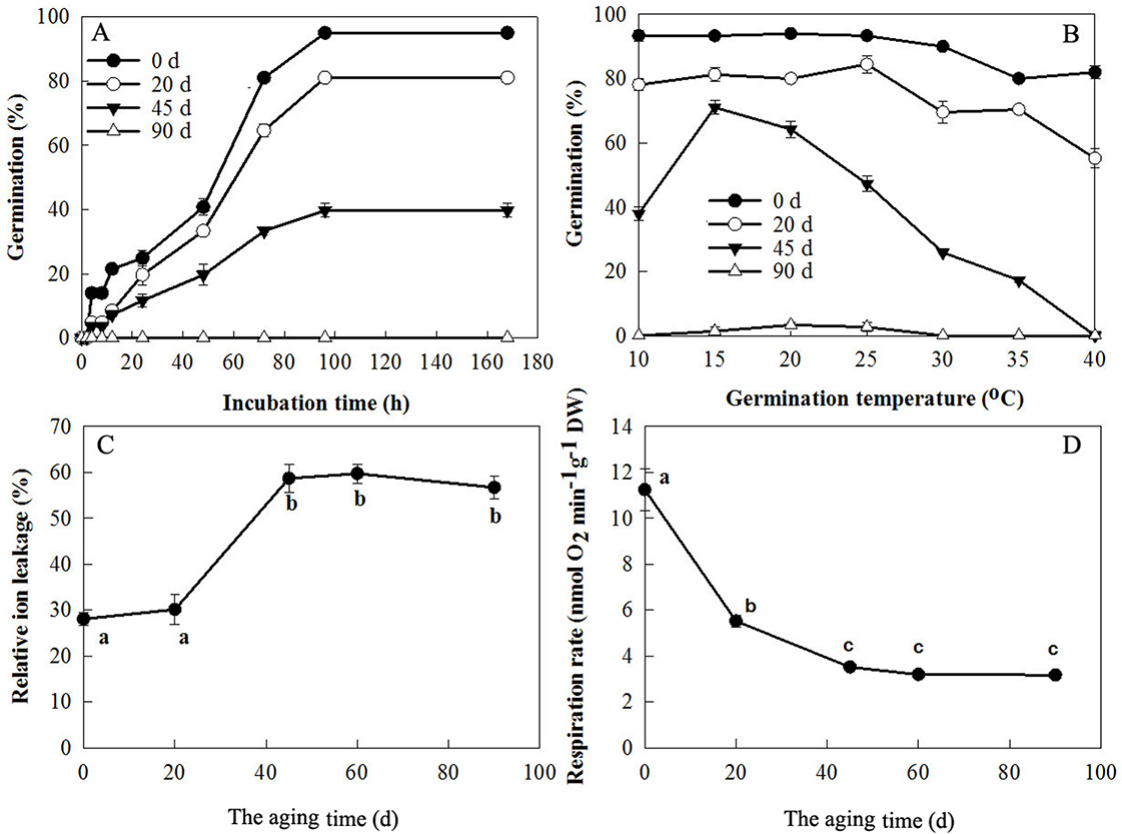
Germination time course (A), response to temperature (B), relative ion leakage (C) and respiration rate (D) of poplar seeds with different vigor. A and B, seeds aged at 30°C and 75% relative humidity for different times were incubated in darkness at 10°C for the indicated time (A) or at 10–40°C for 168 h (B). A radicle protrusion of 1 mm was used as the criterion for completion of germination. C and D, after aging for the indicated time, relative ion leakage and respiration rate of seeds were immediately measured as described in Materials and methods. All values are means ± SD of three replicates of 50 or 100 seeds each. Bars with different lower case letters are significantly different among seeds aged for different times (*P* = 0.05).

Seed vigor is not a single measurable property, but a concept describing several characteristics [1]. Germination percentage, germination rate, relative ion leakage and respiration rate of seeds can be used as parameters to assess seed vigor. Poplar seeds germinated rapidly at 10°C and in darkness. Germination of seeds reached 21% at 12 h of imbibition, 41% at 48 h, and 95% at 96 h (*P* value ≤ 0.001, Fig 1A). The germination percentage and germination rate of aged (low vigor) seeds significantly decreased, for example, the germination percentage of seeds aged for 20, 45 and 90 d was 20, 12 and 0%, respectively, after 24 h of imbibition at 10°C (Fig 1A). We also observed that the temperature range of germination was wider for the control (high vigor) seeds and became narrower for the low vigor seeds. For example, germination of control seeds was above 80% at 10–40°C, while that of seeds aged for 45 d was 71% at 15°C and significantly decreased below and above 15°C (Fig 1B).

Relative ion leakage of seeds was unaffected at 20 d of aging, significantly increased from 20 to 45 d, and maintained a high level after 45 d (Fig 1C). Compared to control seeds, the respiration rate of seeds aged for 20 d decreased by 51%, by 69% for 45 d, and by 75% for 60 and 90 d (Fig 1D).

### The proteome profiles, identification and functional classification of the differentially expressed proteins

**Fig 2:**
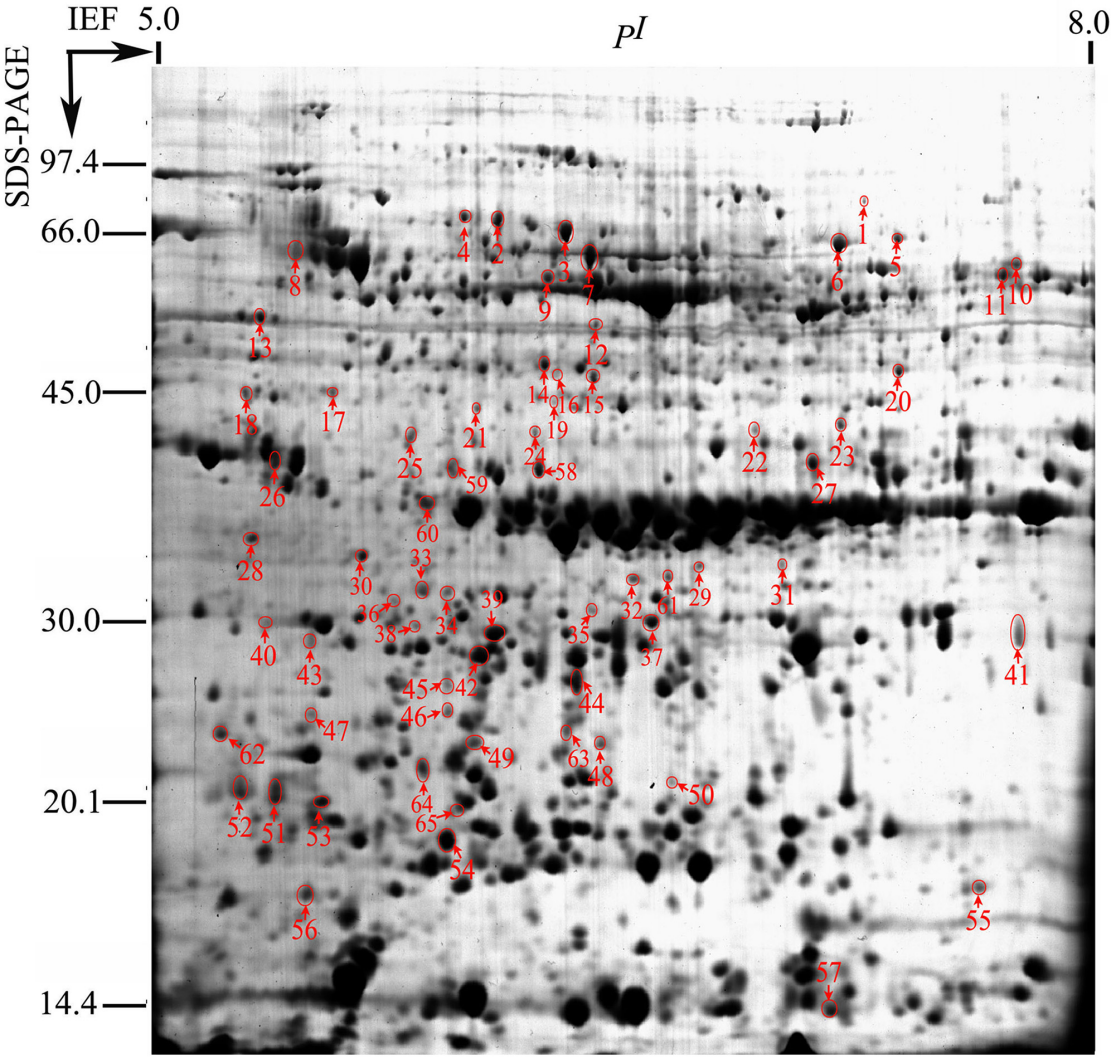
The reference gel (seeds aged for 0 d) for the total protein extract from poplar seeds aged for 0, 45 and 90 d. A total of 600 μg of proteins was extracted from poplar seeds, separated by 2-D gel as described in Materials and methods, and visualized with CBB. The protein spots accumulated differentially in different treatments are numbered and highlighted by circles and arrows. Their identities and properties are described in Table 1.

To investigate further the key events (proteins) associated with seed vigor change, poplar seeds aged for 0, 45 and 90 d were imbibed in water at 10°C for 2 h. No germination was observed in any of these seeds (Fig 1A). After the total soluble proteins were extracted, analyzed by 2-DE and stained by CBB, about 1000 protein spots were detected on each gel (Fig 2, S1 Fig). A total of 81 protein spots showed a significant change of more than 1.5-fold (*P* < 0.05) in abundance when comparing the proteomes among different vigor seeds mentioned above. All of these protein spots were excised and analyzed by MALDI TOF MS/MS, and were identified by first searching in the Swissprot database and, if that showed no matches, then in the NCBI database. Of these 81 protein spots, 65 protein spots were successfully identified (80%).

**Fig 3:**
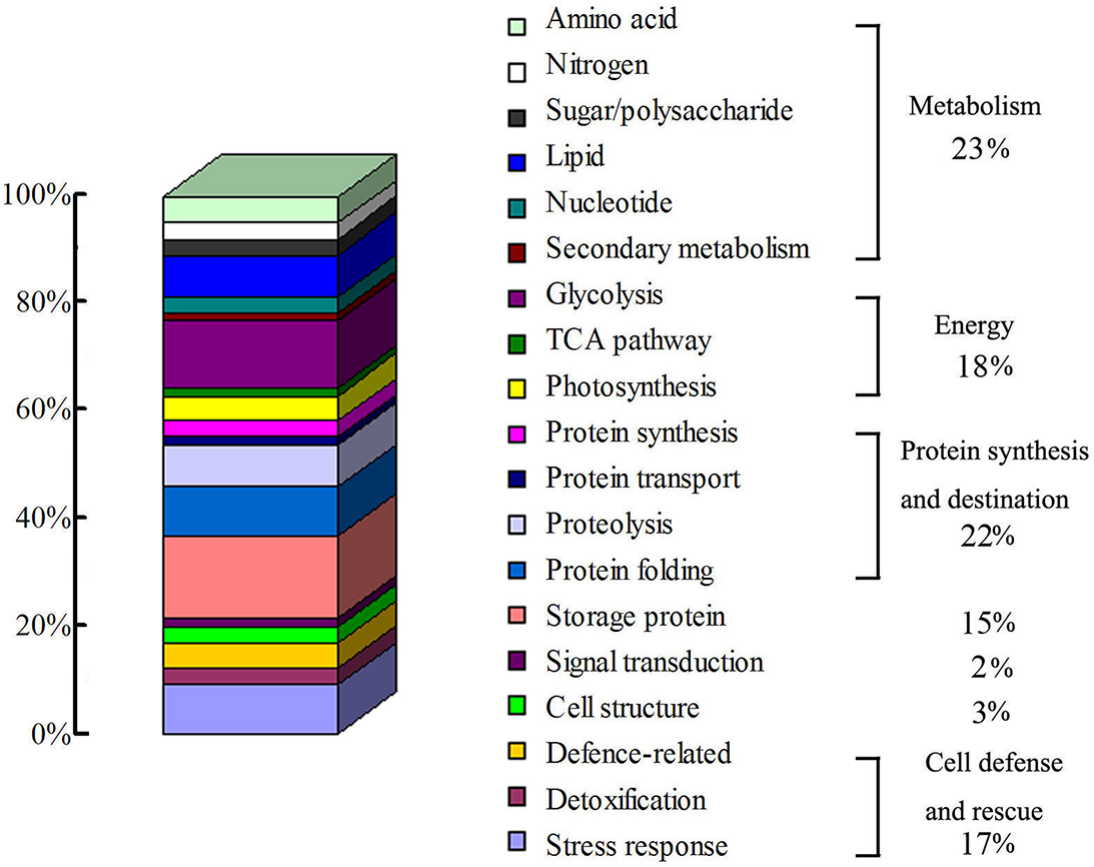
Functional classification and distribution of the 65 proteins differentially changed and identified in poplar seeds aged for 0, 45 and 90 d. These proteins were categorized into 7 functional groups and 19 sub-functional groups according to Bevan et al. [41] and Schiltz et al. [80].

The identified 65 single proteins were matched to 60 unique genes, and could be classified into 7 functional groups and 19 sub-functional groups based upon Bevan *et al*. [41] (Table 1, S1 Table, Fig 3). On the basis of the number of identified proteins, the most numerous functional groups were associated with metabolism (23%), protein synthesis and destination (22%), energy (18%), cell defense and rescue (17%) and storage protein (15%). These proteins accounted for 95% of all the identified proteins (Fig 3).

**Table 1:** Proteins differentially-accumulated and identified by MALDI-TOF-TOF MS in *Populus* × *canadensis* Moench seeds under controlled aging treatment at 30°C and 75% relative humidity for 0, 45 and 90 d. Only protein spots that changed in abundance at least 1.5-fold (*P* < 0.05) of three replicates are included. Some fold changes are between –1.5 and +1.5, because there is a change of at least 1.5-fold in one of the other treatments. The positions of the spots are shown in Fig 2. Exp. protein mass, experimental protein mass; Theo. protein mass, theoretical protein mass. 0, 45 and 90 d, seeds controlled deteriorated at 30°C and 75% relative humidity for 0, 45 and 90 d, respectively; A, appeared; D, disappeared; NA, undetected in both treatments. +, increased;–, decreased. * Blasted from other species.

Biological process	Protein ID	Identified protein name	Accession number	Mascot score	Sequence coverage (%)	Number of sequenced/ matched peptides	Exp. protein mass (kDa)/pI	Theo. protein mass (kDa)/pI	45 d/0 d	90 d/0 d
**Metabolism** (15)										
Amino acid	6	Acetolactate synthase	XP_002322262	110	16	3/8	64/7.1	71/7.2	–2.8	(–1.47)
	14	Glutamate-1-semialdehyde 2,1-aminomutase	XP_002321728	463	26	5/14	48/6.2	52/6.4	+1.59	+1.67
	19	Glutamine synthetase	XP_002301683	160	31	2/6	45/6.2	40/6.0	+2.06	+2.42
Nitrogen	12	Argininosuccinate synthase	XP_002311011	320	30	4/14	52/6.4	55/6.3	+1.85	(+1.22)
	29	Probable pyridoxal biosynthesis protein PDX1	XP_002308219	152	30	2/10	33/6.8	33/6.3	+1.52	+1.73
Sugar/poly- saccharide	21	ATP phosphoribosyltransferase*	XP_002325923	214	20	6/10	44/6.0	46/6.0	+1.67	+1.85
	22	Thymidine diphospho-glucose-4-6-dehydratase	XP_002312082	241	39	6/12	39/6.8	39/6.1	+1.78	+3.48
Lipid	17	GDSL esterase/lipase*	XP_006388670	118	8	1/2	45/5.5	42/5.0	+1.62	+2.02
	28	Inorganic pyrophosphatase*	XP_002298803	123	35	1/9	35/5.3	33/6.6	(+1.31)	+1.57
	32	Hydroxyacylglutathione hydrolase	XP_002329233	67	14	1/2	32/6.5	29/5.9	(+1.21)	+2.04
	36	Inorganic pyrophosphatase*	ABK93990	162	40	3/8	31/5.7	25/5.3	+1.96	(–1.23)
	61	Hydroxyacylglutathione hydrolase	XP_002329233	103	21	2/3	32/6.6	29/5.9	(+1.00)	+1.52
Nucleotide	48	Ribonucleoprotein*	XP_002314019	76	22	1/5	24/6.4	28/9.3	(–1.16)	+1.52
	49	Ribonucleoprotein*	ABK96373	94	6	2/3	24/6.0	28/9.3	+1.82	+2.14
Secondary metabolism	31	Probable pyridoxal biosynthesis protein PDX1	XP_002322981	192	28	2/9	33/7.0	33/6.5	(+1.12)	+1.75
**Energy** (12)										
Glycolysis	2	2,3-Bisphosphoglycerate-independent phosphoglycerate mutase*	XP_002326261	404	24	5/9	66/6.0	61/5.4	+3.39	+2.91
	3	Cytosolic phosphoglucomutase	XP_002311517	152	14	3/6	65/6.3	63/5.5	(–1.29)	–1.73
	4	Phosphoglucomutase*	XP_002311090	106	7	2/3	66/5.9	66/6.2	+1.98	(+1.34)
	9	Enolase*	XP_002322420	640	27	7/9	60/6.2	48/5.7	+5.05	+6.72
	15	Cytosolic phosphoglycerate kinase 1	BAA33801	735	36	8/12	46/6.4	43/5.8	+1.54	+1.75
	16	Phosphoglycerate kinase	XP_002315067	488	32	5/10	46/6.2	43/5.7	+1.73	+1.88
	37	Triosephosphate isomerase	XP_002311168	858	55	8/12	30/6.5	27/6.0	(+1.19)	+1.88
	38	Triosephosphate isomerase	XP_002314179	564	39	5/8	30/5.9	29/5.4	+1.90	+1.56
TCA cycle associated	20	Pyruvate dehydrogenase	XP_002314631	221	14	4/6	46/7.3	44/7.6	+1.82	+1.92
Photosynthesis	18	Magnesium-chelatase subunit chlI	XP_002305090	295	30	5/9	45/5.3	46/5.5	(+1.05)	+1.70
	33	Putative NAD-dependent dehydrogenase 2*	XP_002309064	349	43	4/7	32/5.8	27/5.7	+2.10	+2.33
	55	Peptidyl-prolyl cis-trans isomer	ABK96553	116	19	2/2	17/7.6	16/6.7	(+1.41)	+1.61
**Protein synthesis and destination** (14)										
Protein synthesis	1	Polyadenylate-binding protein*	XP_002301171	270	23	4/13	70/7.2	72/6.2	+2.42	+4.55
	27	Nucleolar protein nop56*	XP_002306784	571	29	7/10	38/7.0	49/5.7	(–1.04)	+1.54
Protein transport	26	NSFL1 cofactor p47*	XP_002317118	116	10	1/1	39/5.4	28/5.4	+1.95	(+1.29)
Proteolysis	13	26S proteasome AAA-ATPase subnit family protein	XP_002319515	428	27	6/11	52/5.3	48/5.0	(–1.14)	+1.55
	34	Proteasome subunit alpha type	XP_002318902	447	42	5/7	32/5.9	26/5.5	(+1.48)	+1.94
	35	Proteasome subunit alpha type	XP_002308263	678	36	10/10	31/6.4	27/5.7	+1.80	+2.10
	46	Proteasome subunit beta type	ADG86642	194	24	2/4	25/5.8	26/5.5	(+1.14)	+1.77
	56	Aspartic proteinase	AAN60260	180	8	2/2	17/5.5	18/4.7	+1.70	+2.56
Protein folding	7	Chaperonin containing t-complex protein 1*	XP_002328218	89	11	1/4	63/6.4	61/6.2	–2.29	–2.38
	24	Protein disulfide isomerase*	ABK93392	298	24	4/6	41/6.2	35/5.3	(+1.41)	+1.62
	40	Groes chaperonin*	XP_002326137	68	8	3/4	30/5.4	27/8.7	+1.67	+3.66
	43	20 kDa chaperonin family protein	XP_002324138	359	29	3/5	29/5.5	27/7.8	(–1.01)	–1.81
	58	Protein disulfide isomerase*	ABK93392	413	16	2/4	38/6.2	35/5.3	(–1.30)	–1.69
	59	Protein disulfide isomerase*	ABK93392	388	20	4/5	38/5.9	35/5.3	+1.65	+2.05
**Storage protein** (10)										
	5	Glutelin type-A 3*	XP_002329509	295	29	3/11	65/7.3	53/6.0	–2.82	(–1.47)
	11	11S globulin seed storage protein 2*	XP_002306851	72	13	2/4	58/7.6	53/6.4	(–1.08)	+1.79
	25	Legumin family protein	XP_002307645	235	15	3/4	42/5.8	54/8.2	(+1.30)	+1.59
	51	Nutrient reservoir*	XP_002313331	304	19	6/10	22/5.4	48/8.3	+2.02	+3.02
	52	Glutelin type-A 3*	XP_002336547	104	5	1/1	22/5.3	56/6.4	+1.53	+1.70
	53	2S albumin family protein	XP_002317577	193	15	2/4	20/5.5	17/4.9	+1.58	+1.62
	60	Glutelin type-A 3*	XP_002336547	124	11	3/5	37/5.8	56/6.4	(+1.45)	+1.63
	63	Legumin family protein	XP_006370926	248	9	2/6	24/6.3	56/6.1	(+1.41)	+2.68
	64	Hypothetical protein POPTR_0019s01840g	XP_002329472	72	7	1/2	22/5.8	56/6.1	(+1.16)	+1.56
	65	Hypothetical protein POPTR_0019s01840g	XP_002329472	484	26	5/12	20/5.9	56/6.1	(–1.39)	+1.58
**Signal transduction** (1)										
	57	Serine/threonine-protein kinase*	XP_002317682	52	56	3/5	14/7.1	13/5.6	+1.85	(–1.04)
**Cell growth and structure** (2)										
Cell structure	39	Dienelactone hydrolase family protein	XP_002312740	436	36	4/7	30/6.0	26/5.5	(+1.18)	+1.58
	54	F5O8.30 protein*	XP_002311776	447	74	4/7	19/5.9	16/5.6	+1.68	+2.42
**Cell defense and rescue** (11)										
Defence- related	42	Phi class glutathione transferase GSTF2	XP_002301942	487	42	5/7	29/5.9	25/5.5	+1.61	+2.57
	41	Zeamatin	XP_002313445	70	10	2/2	30/7.7	27/8.2	–2.15	–3.29
	44	Cysteine proteinase inhibitor	XP_002299179	83	31	1/4	27/6.3	22/5.8	(+1.39)	+1.78
Detoxification	10	Catalase	CAI43948	533	34	6/13	60/7.7	57/6.8	+1.63	+2.18
	23	Aldo/keto reductase*	XP_002319637	530	36	8/11	41/7.1	39/6.2	(+1.16)	+2.26
Stress response	8	Late embryogenesis abundant *	XP_002330137	547	12	2/6	63/5.5	49/5.2	+3.31	+2.65
	30	Late embryogenesis abundant protein D-34*	XP_002328518	376	36	5/6	34/5.5	22/4.9	+1.68	+1.69
	45	Abscisic acid receptor PYR1*	XP_002326522	229	33	4/5	27/5.8	23/5.5	(–1.08)	+1.52
	47	Heat shock factor binding protein*	XP_002331340	65	20	1/1	25/5.5	8/4.3	(+1.11)	+1.89
	50	Universal stress family protein	XP_002324004	265	42	3/8	22/6.6	20/6.0	+1.53	+1.94
	62	Heat shock factor binding protein*	XP_002331340	70	61	1/3	25/5.2	8/4.3	(–1.17)	+1.64

### The proteins differentially accumulated in different vigor seeds

The comparison of differentially accumulated proteins (Table 1, Fig 2) allowed them to be classified into different accumulation patterns (Table 2). Type-1 proteins corresponded to the proteins whose abundance increased by ≥ 1.5-fold at 45 d of aging and stayed high or increased further at 90 d. Type-2 proteins showed a less than 1.5-fold change at 45 d of aging and a ≥ 1.5-fold increase at 90 d. Most of the identified proteins (53 proteins, 82%) belonged to these two types (Tables 1 and 2). Type-3 proteins decreased significantly in abundance at 45 d of aging and stayed low or decreased further at 90 d. Type-4 proteins showed a less than 1.5-fold change at 45 d of aging and a ≥ 1.5-fold decrease at 90 d. Type-3 and -4 proteins accounted for 9% (6 proteins) of identified proteins (Tables 1 and 2). Type-5 proteins (spots 4, 12, 26, 36 and 57) showed a ≥ 1.5-fold increase at 45 d of aging and a less than 1.5-fold change at 90 d of aging; in contrast, type-6 proteins (spot 5) showed a ≥ 1.5-fold decrease at 45 d of aging and a less than 1.5-fold change at 90 d. Type-5 and -6 proteins accounted for 9% of identified proteins (Table 1). Because the accumulation pattern of Type-5 and -6 proteins might not correlate with the loss of seed vigor, these proteins have not been considered in the following analysis.

**Table 2:** Proteins accumulated differentially during aging of *Populus* × canadensis Moench seeds. Only protein spots that changed in abundance at least 1.5-fold (*P* < 0.05) in all three replicates are included. The positions of the spots are shown in Fig 2. Type-1, type-1 proteins corresponded to the proteins whose abundance increased by ≥ 1.5-fold at 45 d of aging and stayed high or increased further at 90 d; Type-2, type-2 proteins showed a less than 1.5-fold change at 45 d of aging and a ≥ 1.5-fold increase at 90 d; Type-3, type-3 proteins decreased significantly in abundance at 45 d of aging and stayed low or decreased further at 90 d; Type-4, type-4 proteins showed a less than 1.5-fold change at 45 d of aging and a ≥ 1.5-fold decrease at 90 d.

Protein function	Accumulation pattern							
	Type-1		Type-2		Type-3		Type-4	
	No	Spot ID	No	Spot ID	No	Spot ID	No	Spot ID
**Metabolism**	**7**		**5**		**1**			
Amino acid	2	14, 19			1	6		
Nitrogen	1	29						
Sugar/polysaccharide	2	21, 22						
Lipid	1	17	3	28, 32, 61				
Nucleotide	1	49	1	48				
Secondary metabolism			1	31				
**Energy**	**7**		**3**				**1**	
Glycolysis	5	2, 9, 15, 16, 38	1	37			1	3
TCA pathway	1	20						
Photosynthesis	1	33	2	18, 55				
**Protein synthesis and destination**	**5**		**5**		**1**		**2**	
Protein synthesis	1	1	1	27				
Proteolysis	2	35, 56	3	13, 34, 46				
Protein folding	2	40, 59	1	24	1	7	2	43, 58
**Storage protein**	**3**	51, 52, 53	**6**	11, 25, 60, 63, 64, 65				
**Cell growth and structure**	**1**		**1**					
Cell structure	1	54	1	39				
**Cell defense and rescue**	**5**		**5**		**1**			
Defence-related	1	42	1	44	1	41		
Detoxification	1	10	1	23				
Stress response	3	8, 30, 50	3	45, 47, 62				
**Total**	**28**		**25**		**3**		**3**	

Type-1 and -2 proteins are mostly involved in metabolism, energy, protein synthesis and destination, storage proteins and cell defense and rescue–they constituted 18, 15, 15, 14 and 15% of the identified proteins, respectively (Tables 1 and 2). Of the Type-3 and -4 proteins, three are involved in protein synthesis and destination (5%), one in metabolism (2%), one in energy (2%), one in cell defense and rescue (2%) (Tables 1 and 2).

Moreover, we observed that 7 protein spots showed a significant change in 45/0 d-aged seeds and not in 90/0 d-aged ones. In contrast, 28 protein spots showed a significant change in 90/0 d-aged seeds and not in 45/0 d-aged ones. Of these 28 protein spots, 7 proteins were found to be involved in protein synthesis and destination, 6 in storage protein, 5 in metabolism, 5 in cell defense and rescue, 4 in energy and 1 in cell growth and structure (Table 1, S1 Table).

## Discussion

### Change in seed vigor and seed proteome during aging of poplar seeds

The vigor of poplar seeds stored (aged) at 30°C and 75% RH gradually decreased as indicated by decreasing germination rate and percentage (Fig 1A), implying that seed vigor is a quantitative trait. This is in accordance with the results of Rajjou et al. [10,42], Xin et al. [28], Wu et al. [27] and Catusse et al. [26]. We also observed that the temperature range for germination of low vigor poplar seeds became narrower (Fig 1B), showing that seeds were damaged by aging and, thereby have a lower tolerance to suboptimal temperatures. Bewley et al. [43] suggested that in dry or partially hydrated seeds (hydration level II), the damage caused by aging is mainly peroxidation of lipids, which results in breakdown of lipids and release of by-products such as reactive aldehydes. Changes in membrane lipids due to peroxidation may be involved in the increase in membrane permeability and ion leakage. An increase of relative leakage from low-vigor poplar seeds (Fig 1C) is consistent with membrane damage. Activation of respiration is one of the key events occurring during seed germination [43–45], and respiration provides the energy and carbon skeleton for seed germination and subsequent seedling growth. The respiration rate decreased rapidly with seed aging (Fig 1D), indicating that mitochondrial structure had been damaged and/or that mitochondrial repair was decreased. However, the increase in ion leakage and the decrease in respiration both levelled off after 45 days, at which point 71% of the seeds were able to germinate. Therefore, the almost complete absence of germination after 90 d of aging is likely to be due to other changes in the seeds.

The type-2 and -4 proteins (Table 2) all showed no significant change during the first 45 d of aging, but a significant change after 90 d. In most cases this significant change was an increase, indicating that protein biosynthesis (or protein modification such as protease degradation of storage proteins) was taking place between 45 and 90 d of aging.

Seven protein spots showed a significant change only in 45/0 d-aged seeds, and 28 protein spots, and only in 90/0 d-aged seeds (Table 1). We therefore think that changes in the poplar seed proteome appearing after 45 and 90 d both contain important information about the loss in seed vigor observed.

### The abundance of proteins involved in amino acid, lipid and nucleotide metabolism increased

Inorganic pyrophosphatase (PPase) hydrolyzes inorganic pyrophosphate to inorganic phosphate, in a highly exergonic reaction. This enzyme plays a critical role in lipid metabolism (including lipid synthesis and degradation) [46]. PPase (spot 28) increased in abundance during aging of poplar seeds (Tables 1 and 2, Fig 2), which might provide more energy for lipid metabolism.

The glyoxalase system is mainly composed of glyoxalase I and II (also known as hydroxyacylglutathione hydrolase, HAGH). Glyoxylase II catalyzes the conversion of *S*-D-lactoylglutathione into D-lactate and glutathione (GSH), thereby reforming the GSH consumed in the glyoxylase I-catalysed step of the glyoxalase pathway [47]. HAGH (spots 32 and 61) increased in abundance during aging of poplar seeds (Tables 1 and 2, Fig 2), thus implicating HAGH in poplar seed vigor.

GDSL esterases/lipases are a newly discovered subclass of lipolytic enzymes that are very important and attractive research objects because of their multifunctional properties, such as broad substrate specificity and regiospecificity. The GDSL esterases/lipases are involved in the regulation of plant development, morphogenesis, synthesis of secondary metabolites, and defense response [48]. GDSL esterase/lipase (spot 17) increased in abundance during aging of poplar seeds (Tables 1 and 2, Fig 2), and its role remains to be investigated.

Ribonucleoproteins (RNPs) play key roles in many cellular processes and often function as RNP enzymes [49]. RNP (spots 48 (XP_002314019) and 49 (62ABK96373)) increased in abundance during aging of poplar seeds (Tables 1 and 2, Fig 2), showing that it might be involved in poplar seed vigor.

Glutamate-1-semialdehyde aminomutase (GSAM) is a dimeric, pyridoxal 5'-phosphate-dependent enzyme in plants and some bacteria catalyzing the isomerization of L-glutamate-1-semialdehyde to 5-aminolevulinate, a common precursor of chlorophyll, haem, coenzyme B_12_, and other tetrapyrrolic compounds [50]. Glutamine synthetase assimilates ammonium into glutamine, which provides nitrogen groups, directly or via glutamate. As the first enzyme of the nitrogen assimilatory pathway, glutamine synthetase plays a regulatory role in nitrogen metabolism and plant productivity [51]. GSAM (spot 14) and glutamine synthetase (spot 19) increased in abundance during aging of poplar seeds (Tables 1 and 2, Fig 2), but the role of these two proteins in seed vigor is not clear.

ATP-phosphoribosyl transferase (ATPPRT), which catalyzes the first committed step of histidine (His) biosynthesis, is feedback inhibited by the pathway end-product, L-His [52,53]. Thymidine diphosphate glucose (TDP-glucose) is a nucleotide-linked sugar consisting of deoxythymidine diphosphate linked to glucose. It is the starting compound for the syntheses of many deoxysugars [54]. ATPPRT (spot 21) and TDP-glucose-4-6-dehydratase (spot 22) both increased in abundance during aging of poplar seeds (Tables 1 and 2, Fig 2), but the effect of these two proteins on seed vigor is also not known.

Acetolactate synthase is a common enzyme that catalyzes the first step of the biosynthetic pathway of the branched-chain amino acids, valine, leucine and isoleucine [55]. Acetolactate synthase (spot 6) decreased in abundance with aging of poplar seeds (Tables 1 and 2, Fig 2), indicating that biosynthesis of branched-chain amino acids is important for seed vigor.

### Seed aging is an energy-dependent process

Triosephosphate isomerase (TPI), phosphoglycerate kinase (PGK), phosphoglycerate mutase (PGM) and enolase all catalyze steps in glycolysis, an important pathway providing energy and carbon skeleton to intermediary metabolism, which takes place in the cytosol of plant cells [56]. Pyruvate dehydrogenase (PDH), a key enzyme forming the entry point into the tricarboxylic acid cycle, converts pyruvate into actyl-CoA and NADH [56]. We observed that TPI (spots 37 and 38), PGK (spot 16, XP_002315067), cytosolic PGK1 (spot 15, BAA33801), 2,3-bisphosphoglycerate-independent PGM (spot 2), enolase (spot 9) and PDH (spot 20) all increased in abundance in aged poplar seeds (Tables 1 and 2, Fig 2), indicating that the loss in seed vigor is an energy-dependent process involving the glycolytic pathway and perhaps also the tricarboxylic acid cycle. However, this picture is complicated by the expression pattern of cytosolic phosphoglucomutase (spot 3), yet another glycolytic enzyme, which showed no significant change at 45 d of aging, and was significantly decreased at 90 d of aging, a pattern opposite to the other glycolytic enzymes mentioned above (Tables 1 and 2, Fig 2).

### Changes in proteins involved in protein synthesis and destination

Polyadenylate-binding proteins (PABPs) and nucleolar protein nop56 involved in protein synthesis increased in abundance. PABPs are found throughout eukaryotes, and bind the poly(A) tails of mRNAs through N-terminal RNA recognition motifs. PABPs also interact with eukaryotic initiation factors associated with the 5'-cap of mRNA, resulting in mRNA circularization, which favors initiation of translation and ribosome recycling. It is well known that PABPs play a variety of roles, including regulation of mRNA translation and stability. Most PABPs contain a conserved C-terminal domain, which binds proteins that can modulate PABP function [57]. Nucleolar protein nop56 is a member of the core proteins of box C/D snoRNP complexes that direct 2'-*O*-methylation of pre-rRNA ribose moieties during the early stages of pre-rRNA processing. Genetic analysis of yeast Nop56 indicates that it is responsible for cleaving 35S pre-rRNA to produce 25S rRNA (the mature rRNA corresponding to human 28S rRNA) [58]. PABP (spot 1) and nucleolar protein nop56 (spot 27) increased in abundance (Tables 1 and 2, Fig 2), indicating that protein synthesis de novo might be necessary during aging of poplar seeds.

Protein disulfide isomerases (PDI) increased in abundance while chaperonin containing t-complex protein 1 associated with protein folding decreased. PDIs typically catalyse the formation and isomerization of disulphide bonds during the folding of nascent proteins. Some PDIs may also have chaperone roles under house-keeping and/or stress conditions [27]. During aging of poplar seeds, the abundance of spot 58 (PDI) decreased, whereas spots 24 and 59 (PDI) increased (Tables 1 and 2, Fig 2). PDIs abundance, taken together, increased during aging. Chaperonin containing t-complex protein 1 (spot 7) decreased in abundance during aging of poplar seeds (Tables 1 and 2, Fig 2), implying a decreasing protein repair capacity. Groes chaperonin is present in all three kingdoms of life and is one of the most conserved proteins in living organisms. The conjunction of Groes chaperonin with chaperonin 60 forms a protein-folding machine required for correct folding of many proteins and for recycling of misfolded proteins [59]. Groes chaperonin was encoded by two different genes (XP_002326137 and XP_002324138), and spot 40 increased and spot 43 decreased in abundance (Tables 1 and 2, Fig 2), indicating that Groes chaperonin has different roles in aging of poplar seeds.

### Increased protein degradation is associated with seed aging

Truncated storage proteins increased in abundance during aging. Seeds contain mainly three kinds of stored reserves, storage proteins, lipids and starch. A number of different storage proteins–legumin family protein (spots 25 and 63), 11S globulin seed storage protein 2 (spot 11), nutrient reservoir (spot 51), glutelin type-A 3 (spots 52 and 75) and 2S albumin family protein (spot 53) increased in abundance in low-vigor poplar seeds (Tables 1 and 2, Fig 2). With one exception, the experimental size of these proteins was much smaller than their theoretical size (Table 1, Fig 2), indicating that they derived from protein degradation. The exception was 11S globulin seed storage protein 2 (spot 11), which appeared at the right size, but a substantially higher pI (7.6 against 6.4 for the gene product), probably caused by a posttranlationally modification other than proteolytic degradation. At the same time, only one of the major protein spots containing a full-sized storage protein (spot 5, glutelin type-A3) decreased significantly in size, indicating that in the majority of cases only a minor part of the storage proteins had been modified.

We conclude that the loss of seed vigor is associated with active protein degradation, but it is apparently not caused by a shortage of storage proteins.

Proteasome and aspartic proteinase related to proteolysis showed high accumulation. In all eukaryotic cells, the 26S proteasome plays an essential role in the process of ATP-dependent protein degradation. The proteins targeted for degradation by the 26S proteasome are first tagged by covalent linkage of a polyubiquitin chain, and are then recognized by the 26S proteasome and rapidly degraded into short peptides. It has been proposed that this ubiquitin-mediated protein degradation pathway plays the primary role in the degradation of most short-lived proteins involved in cell cycle, transcriptional regulation, apoptosis, as well as in the degradation of abnormal proteins such as misfolded and damaged proteins [60]. Aspartic proteinases are a family of proteolytic enzymes that are widely distributed in nature and are involved in several physiological processes in plants, including protein processing, senescence, and stress response [61]. Proteasome subunit alpha type (spots 34 (XP_002318902) and 35 (XP_002308263)), proteasome subunit beta type (spot 46), 26S proteasome AAA-ATPase subnit family protein (spot 13) and aspartic proteinase (spot 56) increased in abundance during aging of poplar seeds (Tables 1 and 2, Fig 2), which indicated that protein degradation increased with seed aging.

### Changes in protein involved in cell defense and rescue

Phi class glutathione transferase GSTF2 increased in abundance. The plant-specific phi class of glutathione transferases (GSTFs) are often highly stress-inducible and expressed in a tissue-specific manner, suggesting that they have important protective roles [62]. Phi class glutathione transferase GSTF2 (spot 42) increased in abundance during aging of poplar seeds (Tables 1 and 2, Fig 2), which would help the seeds to protect themselves against aging damage.

Catalase and aldo/keto reductase increased in abundance. Reactive oxygen species (ROS), e.g., O_2_^−^ and H_2_O_2_, are continuously synthesized in cells, even under unstressed conditions [63]. ROS can attack lipids, proteins and DNA, and result in lipid and protein peroxidation and DNA damage [64]. Rajjou et al. [42] demonstrated that protein oxidation (carbonylation) strongly increased in aged seeds. Catalase converts H_2_O_2_ into O_2_ and H_2_O [65,66]. Aldo/keto reductase is an NADPH-dependent oxidoreductase, which primarily reduces non-protein aldehydes and ketones to alcohols [67]. Aldo/keto reductses have specific role in the detoxification of a wide range of exogenous and endogenous compounds including methylglyoxal formed from the glycolytic pathway and lipid peroxidation [68]. Catalase (spot 10) and aldo/keto reductase (spot 23) increased in abundance during aging of poplar seeds (Tables 1 and 2, Fig 2), indicating that these two enzymes are involved in detoxification in aged poplar seeds.

Late embryogenesis abundant (LEA) proteins and heat shock proteins (HSPs) are potential low seed vigor markers. LEA proteins accumulate during seed development, coincident with acquisition of desiccation tolerance of the developing seeds, and disappear during germination. They are presumed to be involved in binding or replacement of water, in sequestering ions that will concentrate under dehydration conditions, or in maintaining protein and membrane structure [69]. LEA protein (spot 8) and LEA protein D-34 (spot 30) increased in abundance during aging of poplar seeds (Tables 1 and 2, Fig 2). This is consistent with the results of Catusse et al. [26] and Yacoubi et al. [29], who found that LEA D-34 and LEA 35 kDa seed maturation protein increased in abundance in aged sugarbeet seeds and decreased in abundance in primed and aged-primed alfalfa seeds. These results are in accordance with the finding that LEA proteins constitute low seed vigor markers as proposed for Arabidopsis [42], soybean [70], beech [71] and sugarbeet [26]. HSPs act as molecular chaperones, repairing and aiding the renaturation of stress-damaged proteins, and in that way help protecting cells against stress [72,73]. We observed that heat shock factor binding protein (HSFBP, spot 62) increased in abundance during aging of poplar seeds (Tables 1 and 2, Fig 2). Yacoubi et al. [29] proposed that HSPs are a potential seed vigor marker. Prieto-Dapena et al. [74] reported that transgenic Arabidopsis seeds overaccumulating a heat stress transcription factor exhibited enhanced accumulation of HSPs and improved resistance to aging. We also noted that HSFBP (spots 47 and 62) increased in abundance in low vigor poplar seeds (Tables 1 and 2, Fig 2).

Furthermore, zeamatin is a protein isolated originally from *Zea mays* and has antifungal activity against human and plant pathogens. Unlike other pathogenesis-related group 5 proteins, zeamatin inhibits insect α-amylase and mammalian trypsin activities [75]. Zeamatin (spot 41) decreased during aging of poplar seeds (Tables 1 and 2, Fig 2), showing a decreasing antifungal activity with aging. Cysteine proteinase inhibitors (CysPIs), which specifically inhibit sulfhydryl proteinase activities, are distributed ubiquitously among animal, plant, and microorganism species. Two physiological functions of CysPI have been proposed: regulation of protein turnover and host plant defense against insect predation and, perhaps, pathogens [76]. CysPI (spot 44) increased in low vigor poplar seeds (Tables 1 and 2, Fig 2). It has been proposed that there are three CysPI genes in soybean leaves, CysPI gene (*L1*) was constitutive and the other two (*N2* and *R1*) were induced by wounding or methyl jasmonate treatment, and induction of *N2* and *R1* transcript levels in leaves occurred coincidentally with increased papain inhibitory activity [76].

The phytohormone abscisic acid (ABA) mediates the adaptation of plants to environmental stresses such as drought and regulates developmental signals such as seed maturation, dormancy and germination. In plants, the PYR/PYL/RCAR family of START proteins receives ABA to inhibit the phosphatase activity of the group-A protein phosphatases 2C, which is major negative regulators in ABA signaling [77,78]. ABA receptor PYR1 (spot 45) increased in low vigor poplar seeds (Tables 1 and 2, Fig 2), which may point at an increasing ABA response with aging.

## Conclusions

Changes in the proteome profile of different vigor poplar seeds were monitored and that led to the identification of a number of candidate proteins associated with change of seed vigor. The decrease in seed vigor (aging) is directly associated with proteins involved in metabolism (acetolactate synthase, PPase, HAGH and RNP), energy (TPI, cytosolic PGK1, 2,3-bisphosphoglycerate-independent PGM, enolase, PDH and cytosolic phosphoglucomutase), protein synthesis and destination (PABP, nucleolar protein nop56, proteasome, aspartic proteinase, PDI) and cell defense and rescue (Phi class glutathione transferase GSTF2, catalase, aldo/keto reductase). It appears that the decrease in seed vigor is an energy-dependent process, which requires protein synthesis and degradation as well as cellular protection. Seed vigor is a complex trait involving many biochemical and molecular processes [42,79]. Proteome analyses of seed vigor are very useful for understanding the molecular mechanism of seed vigor change and increasing seed quality.

## Supporting Information

S1 Fig 2D gels of proteins extracted from poplar seeds aged for 0, 45 and 90 d. Supporting Information Available: This material is available free of charge via the Internet at http://www.uniprot.org/.
(TIF)

S1 Table Accumulation levels, accumulation ratios and associated *P* values of proteins listed in Table 1. (XLS)
