# Supplementary figures and images for: Proteome Analysis of Poplar Seed Vigor
Source: PLoS One. 2015 Jul 14;10(7):e0132509. doi: 10.1371/journal.pone.0132509 (PMC4501749; doi:10.1371/journal.pone.0132509)

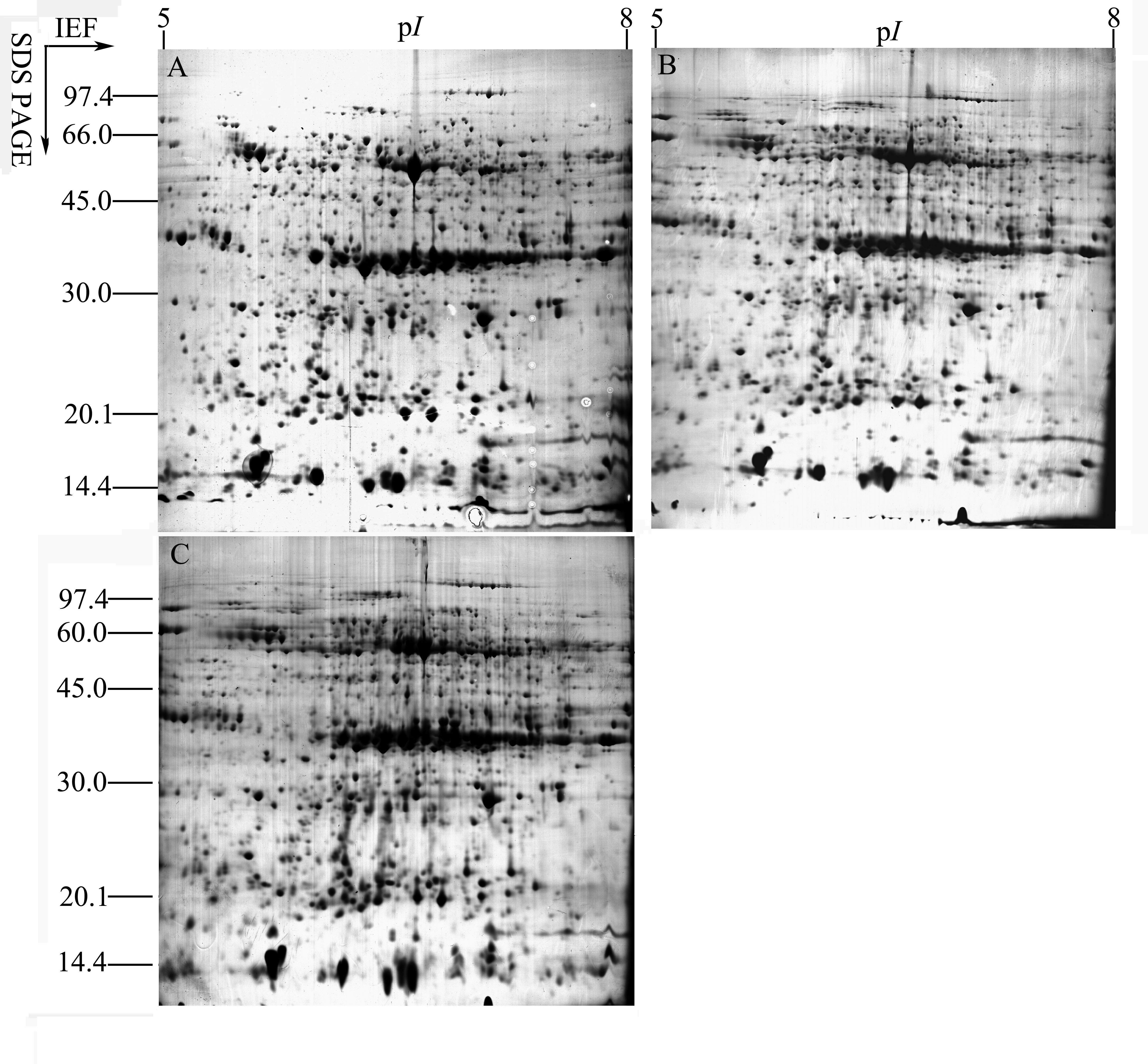

Supplement: S1 Fig — Supporting Information Available: This material is available free of charge via the Internet at http://www.uniprot.org/. (TIF) [file pone.0132509.s001.tif]
